# Retracted: The Immune System in Hepatocellular Carcinoma and Potential New Immunotherapeutic Strategies

**DOI:** 10.1155/2016/2514067

**Published:** 2016-03-31

**Authors:** BioMed Research International

The article titled “The Immune System in Hepatocellular Carcinoma and Potential New Immunotherapeutic Strategies” [1] has been retracted as it was found to contain a substantial amount of material, without referencing, from the following published article: “Potential of immunotherapy for hepatocellular carcinoma,” by Ekaterina Breous and Robert Thimme, in Journal of Hepatology. The author list has been corrected to remove Salvatore Gruttadauria, Isidoro Di Carlo, Adriana Toro and Alessandra Mangia, who were not involved in the preparation or submission of this article and should not have been listed as authors. The first author, Gaetano Bertino, accepts full responsibility and apologizes to the journal and the other authors. The rest of the authors are not culpable in this plagiarism and are victims of this action.

## References

[1] Bertino G., Demma S., Ardiri A. (2015). The immune system in hepatocellular carcinoma and potential new immunotherapeutic strategies. *BioMed Research International*.

